# Thwarting amyloidosis: IL-17 as a disease modifier along the gut/brain axis

**DOI:** 10.1172/JCI194443

**Published:** 2025-07-01

**Authors:** Wade K. Self, David M. Holtzman

**Affiliations:** Department of Neurology, Hope Center for Neurological Disorders, Knight Alzheimer’s Disease Research Center, Washington University School of Medicine, St. Louis, Missouri, USA.

## Abstract

Recent studies have highlighted a possible role for gut microbiota in modulating Alzheimer’s disease pathology, particularly through the actions of gut-derived metabolites and their influence on the immune system. In this issue of the *JCI*, Chandra et al. reveal that circulating levels of the gut microbiota–derived metabolite propionate affected amyloid burden and glial activation in a mouse model of Aβ amyloidosis. The study also identifies a mechanism for the therapeutic benefit of propionate supplementation, showing that propionate lowered peripheral IL-17 and suppressed Th17 cell activity. These results support the idea of therapeutic targeting of the gut/brain/immune axis, particularly via modulation of Th17 responses, and suggest translational strategies involving microbiome-based or immunological interventions for dementia prevention and treatment.

## A gut/immune/brain axis in Alzheimer’s disease

There is a growing interest in targeting regulators of innate and adaptive immunity for the treatment of Alzheimer’s disease (AD) (1). The gut microbiota are important regulators of the immune system, and nearly a decade ago, studies showed that altering the gut microbiota with early-life antibiotics (ABX) treatment, or rearing animals in gnotobiotic conditions, reduced amyloid-β (Aβ) pathology in transgenic mouse models of Aβ amyloidosis (2, 3). In addition, observations of altered gut microbiome composition in humans with clinical (4) or preclinical AD (5) compared with nondiseased individuals have sparked significant interest in the potential of therapeutically targeting the gut/brain axis to modify AD pathogenesis.

However, a central paradox has emerged from preclinical studies in transgenic models: how can findings from gnotobiotic or ABX-treated mice be translated meaningfully to human contexts, since a healthy gut microbiota community is instrumental in the growth and development of the host organism? Findings from studies that reintroduced short-chain fatty acids (SCFAs), a known class of gut microbiota–derived metabolites, into mouse models of Aβ amyloidosis epitomize this translational challenge. These microbial metabolites, produced through the fermentation of dietary fiber, are generally regarded as beneficial for host physiology (6). However, when introduced into mice in gnotobiotic (7) and specific pathogen–free (SPF) conditions (8), some SCFAs appear to promote amyloid accumulation, indicating a complex dynamic, wherein the very metabolites underpinning a healthy symbiotic relationship may also contribute to pathological processes under certain conditions.

**Figure 1:**
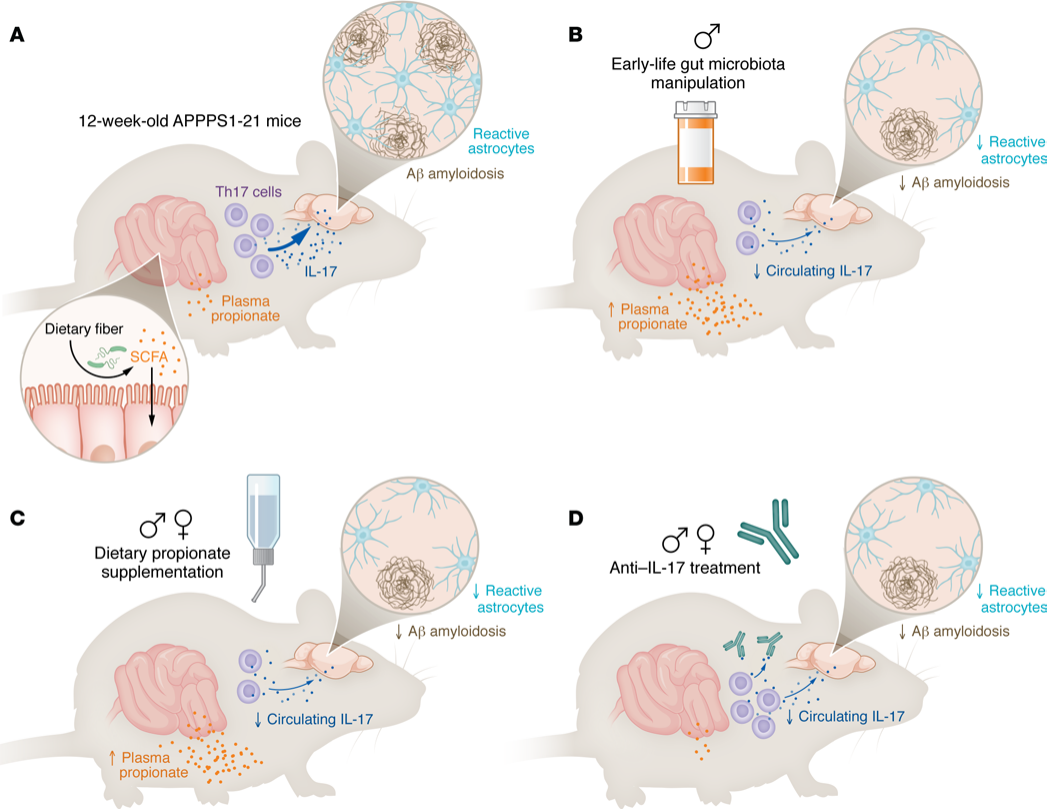
A gut/immune/brain axis involving propionate and IL-17 modifies amyloid accumulation in a mouse model of Aβ amyloidosis. (**A**) Aβ deposition and reactive astrocytosis are observed in the cortex of 12-week-old APPPS1-21 mice, with low levels of propionate in circulation. (**B** and **C**) Increasing circulating levels of propionate via early-life ABX treatment in male mice or supplementation of propionate in drinking water in both male and female mice results in reduced amyloid pathology and astrocyte activation. Increases in propionate coincide with decreases in circulating Th17 cells and IL-17 in the periphery. (**D**) Depletion of circulating IL-17 with a monoclonal antibody phenocopies the effects observed with propionate supplementation.

In this issue of the *JCI*, Chandra et al. provide multiple lines of evidence that a key target of the gut/brain axis involved in modulating brain Aβ amyloidosis may be the effect of a specific SCFA, propionate, on reducing IL-17 in the peripheral circulation (Figure 1) (9). Combined with other studies that have demonstrated the association of propionate and IL-17 in modulating pathologies that contribute to dementia and other neuroimmune disorders (10–12), these findings support the potential utility of modifying the peripheral immune system to treat or prevent dementia and indicate that understanding an individual’s gut microbiota composition and function will be important in developing such strategies.

## Peripheral propionate inversely correlates with Aβ plaque deposition

Chandra et al. (9) began by applying their established broad-spectrum ABX treatment paradigm to APPPS1-21 mice for 1 week before weaning, a model in which they had previously observed reductions in amyloid accumulation, microgliosis, and astrogliosis in male mice, but not in female mice (13, 14). In the current study, they used targeted mass spectrometry and found that only male mice exhibited increased levels of circulating propionate following ABX treatment at 12 weeks of age, the time point at which brain pathology was assessed (9). This finding aligned with earlier metagenomic characterizations that identified an increase in *Akkermansia*, a known propionate-producing bacteria genus, in male mice only (14). Excitingly, Chandra et al. (9) showed that supplementation with 150 mM propionate in mice with an intact gut microbiota successfully replicated the ABX-associated effects of reduced amyloid accumulation and astrocytosis in both male and female APPPS1-21 mice. These data suggest that one of the reasons that female APPPS1-21 mice in this model do not have lowered amyloid accumulation is that there is a differential response in female mice to the short-term ABX protocol, rather than a differential response to gut-derived metabolites like propionate. These results provide great promise that the mechanisms by which propionate mediates responses to brain Aβ deposition will have translational relevance in both males and females, but indicate that sex must be a consideration for any intervention that aims to manipulate gut microbiota composition and function for therapeutic benefit.

## SCFA effects depend on dose, composition, and model

It is important to note that the concentrations of propionate used in this study were much higher than physiological plasma propionate levels in both mice and humans (typically in the micromolar range) (15). High-dose SCFA supplementation in drinking water protocols were originally developed to mimic the concentration of these metabolites in the colonic lumen to study the effects of SCFAs on Treg function in gnotobiotic mice (16), and this approach has yielded conflicting results in SPF and gnotobiotic models of Aβ amyloidosis. For instance, Erny et al. demonstrated that supplementation with a mixture of acetate (67.5 mM), butyrate (40 mM), and propionate (25.9 mM) (17), or acetate alone (150 mM) (7), was sufficient to restore microglial gene expression profiles, morphological features, and innate immune responses toward viral infection in nontransgenic animals, and to restore Aβ amyloidosis in a gnotobiotic 5XFAD-transgenic mouse model similar to that observed in SPF conditions. Colombo et al. also observed an increase in Aβ deposition in APPPS1-21 mice in both SPF and gnotobiotic conditions using the same SCFA mixture of acetate (67.5 mM), butyrate (40 mM), and propionate (25.9 mM) (8). It is possible that the contrasting finding by Chandra et al. (9) — reduced amyloid in response to one SCFA — is, first, related to differences between gnotobiotic mice with immature immune systems compared with SPF mice that have life-long exposure to microbes. Second, no other group has tested the effect of such a high dose (150 mM) of propionate in SPF conditions. In addition, no other group has tested the effect of such a high dose (150 mM) of propionate. However, the findings from this study would be strengthened if it was confirmed that high doses of acetate or butyrate do not mediate the same effect.

Notably, Chandra et al. (9) focused many of their histological and sequencing analyses on astrocyte responses, and this group previously demonstrated that features of plaque-associated astrocytosis are altered with the short-term ABX paradigm in the presence or absence of microglia (14). However, given the clear role for microglia in Aβ deposition and the crosstalk between the two cell types in response to disease pathology (18), it could be helpful to further study whether different SCFA combinations differentially affect all glial cell types in the brain in SPF conditions. This is especially noteworthy, considering that the group’s single-nucleus RNA-Seq dataset showed that the second-most affected cell type in terms of differentially expressed genes was the oligodendrocyte (9).

## A mechanism for translation

Despite remaining questions about the underlying mechanisms, Chandra et al. (9) provide clear evidence that increasing circulating levels of propionate have a therapeutic effect on the brain in responding to Aβ deposition. In considering the translation of this strategy to humans, it would be helpful to know the absolute concentration changes in plasma propionate that were observed after short-term ABX treatment, as the group only reported relative abundances of all metabolites in their targeted assay. These data would enable researchers to design studies to match that level with the dose of propionate in drinking water and to develop probiotic strategies to increase the abundance of certain species of gut bacteria to achieve therapeutic levels of circulating propionate. Given the group’s metagenomic data, *Akkermansia* would be an interesting candidate to test this hypothesis and strategy.

A propionate supplementation strategy to modify brain pathophysiology and/or comorbidities that increase dementia risk is supported by a recent proof-of-concept human study involving individuals with multiple sclerosis (MS) (10). In that study, Duscha et al. showed lower fecal and circulating plasma levels of propionate in patients with MS. Supplementation of MS patients with 1 gram of propionate per day, a dose approximately 200 times lower than what Chandra et al. (9) tested in mice, resulted in reductions in the relapse rate and disease progression over a 3-year follow-up period that coincided with decreases in circulating Th17 cells and altered gut microbiome composition, with changes in immune responses observed only 14 days after the start of treatment (10).

Chandra et al. (9) appear to converge on a similar mechanism in their studies, as they demonstrate that ABX treatment in male mice, or propionate supplementation in either sex, resulted in decreases in circulating Th17 cells and IL-17. They also show that peripheral IL-17 antibody administration to APPPS1-21 mice was sufficient to reduce Aβ deposition and astrocytosis in the brain. By reanalyzing a previously published human AD PBMC dataset, the authors demonstrate increases in peripheral Th17 cells in patients with AD compared with controls (19), supporting the translational relevance of their findings. This work adds to a growing body of evidence in preclinical models that altering Th17 cell abundance or function, either directly or by increasing Treg function, has the ability to modify AD-associated pathologies (20–22) or comorbidities associated with dementia (11, 12). Similarly, clinical studies indicate increased levels of IL-17 in cerebrospinal fluid (CSF) or an imbalance of circulating Th17 and Tregs in individuals with dementia (23, 24). Given the lack of data on any Th17-related biomarkers in the CSF of any mice used in these studies, an outstanding question is whether an opportunity exists to further lower amyloidosis and astrocytosis by targeting IL-17 in both the periphery and CNS.

## Conclusions and future directions

Overall, the results reported by Chandra et al. (9) contribute to a growing rationale for targeting Th17 cells and IL-17 production in the periphery as part of therapeutic strategies for dementia and indicate that gut microbiota–based interventions should strive to modify this pathway. Excitingly, multiple FDA-approved IL-17 monoclonal antibodies are available for the treatment of psoriasis, and the slightly higher associated risks of all-cause dementia in individuals with psoriasis (25, 26) provide motivation to possibly repurpose these compounds in therapeutic strategies for dementia. Ongoing clinical trials are also examining how fiber supplementation may help augment IL-17 therapies for autoimmune disease (NCT06055699, NCT05812157), and the data in this report suggest that similar approaches that take into consideration an individual’s gut microbiome status should be incorporated to maximize possible therapeutic benefits in the setting of dementia pathophysiology.

## Version 1 07/01/2025

Electronic publication
